# The Family as an Actor in High School Students’ Eating Habits: A Qualitative Research Study

**DOI:** 10.3390/foods9040419

**Published:** 2020-04-03

**Authors:** Almudena Garrido-Fernández, Francisca María García-Padilla, José Luis Sánchez-Ramos, Juan Gómez-Salgado, Elena Sosa-Cordobés

**Affiliations:** 1 Department of Nursing. Nursing School, University of Huelva, 21007 Huelva, Spain; almudena.garrido@denf.uhu.es (A.G.-F.); fmgarcia@denf.uhu.es (F.M.G.-P.); jsanchez@denf.uhu.es (J.L.S.-R.); 2 Department of Sociology, Social Work and Public Health. Labour Sciences School, University of Huelva, 21007 Huelva, Spain; 3 Safety and Health Postgraduate Programme, Universidad Espíritu Santo, 092301 Guayaquil, Ecuador; 4 Doctoral Programme, Nursing School, University of Huelva, 21007 Huelva, Spain; elenasosacordobes@gmail.com

**Keywords:** nutrition and health, food sociology, adolescents, school, family, eating habits

## Abstract

In order to discover family conceptions and their difficulties with regard to healthy eating habits during the school day, a qualitative study framed in the phenomenological, exploratory, and explanatory perspective has been carried out to detect and describe the aspects and interrelationships that shape the study phenomenon. The researchers performed triangulation techniques and information analysis support with the Atlas-ti programme. As participants, the students’ parents belonging to public secondary education high schools in Huelva, and the capital and its province were included. The participants were intentionally chosen based on established selection and segmentation criteria. Four main categories were obtained from the triangulated analysis. Healthy breakfast, school snack, school cafeteria, and promotion of healthy food measures. Other subcategories were established within them. Families are well aware of the composition of a healthy breakfast, although this is often not translated into practice. Lack of time, comfort and market influence are the main challenges they encounter for their children to acquire healthy habits. The maintenance of healthy habits, their responsibility and control on behalf of the family, and promoting fruit consumption and healthy products from the part of the centre and its cafeteria were highlighted as improvement proposals.

## 1. Introduction

Regular physical activity (PA) and an adequate diet are essential to improve health-related quality of life [1].

Approximately four out of ten young Spaniards are exposed, from a very early age, to cardiovascular risk factors, with negative short/long-term effects, which are potentially modifiable [2].

From very early stages, the child acquires certain eating habits within its family and social nucleus [3]. Strategies for acquiring healthy habits and modifying harmful habits at this age range are essential to improve health in the adolescent population and in the future adult population [4,5].

Some studies evidence that infant and adolescent diet during the school day is characterised by the elimination of breakfast or an insufficient intake, supplemented with products which are not recommended in a healthy diet, and many products being purchased at the school cafeteria [6,7,8].

The most worrying consequence of these habits is childhood obesity, whose prevalence has been increasing in recent years [2,9].

Within the school context, there is low protection of a healthy eating environment: low quality food supply at school cafeterias, external premises that favour the consumption of unhealthy products, and the absence of promotional activities [8,10]. The school environment should promote more appropriate eating habits. However, in adolescence, eating during the school day is marked by excess food and products of poor nutritional quality [6] Family and schools should be active in promoting healthy eating [11].

Parental health-promoting behaviours are related to what their adolescent children practice [12,13,14,15,16,17], especially breakfast habits [18,19,20,21,22,23,24,25,26,27]. Parents also have an active role in the prevention of certain non-desired habits regarding food consumption [28].

The socialisation role of families regarding the acquisition of healthy eating habits is based on the same family eating habits and the style of parents’ childcare [28,29].

Parents are the people responsible of preparing meals, and they directly influence choices when shopping for food and preparing meals [30,31]. They are important socialisation agents, role models in their children’s lives, and they transmit rules, knowledge, attitudes and behaviours to their children [28,29,30,31,32].

In the literature analysis, we find that most studies relate better eating habits and the consumption of a healthier breakfast with a higher education, economic and social level of families [4,16,19,21,27], and these studies also relate skipping breakfast and dinner as a family with a higher consumption of non-healthy food [13,14,15,26].

It seems that carrying out educational interventions aimed at the promotion of healthy eating in adolescents has positive effects, and that these are even better if families, teachers and healthcare professionals are involved in the process [1].

However, we have not located studies that have worked on the role the family has in their children’s diet (referring to adolescents) during the school day, so we consider it important to open new lines of research that incorporate this subject and complement it with the school area, both as promoters of health care, as the purpose of the study is to improve eating habits among Secondary Education students during the school day, with the help of families, by providing them with resources and abilities aimed at the promotion of healthy eating habits, as well as with strategies to facilitate this task, for example, involving schools and parents.

Our goal has been to describe family conceptions and difficulties about healthy eating during the school day and to know the proposals towards improving healthy eating habits in their children.

This study is framed within the phenomenological theory and is designed by following the classical structure of scientific articles display (Introduction, Material and Method, Results, Discussion, and Conclusion).

## 2. Materials and Methods

### 2.1. Design

Qualitative study framed in the phenomenological perspective. Exploratory study towards knowing the practices and experiences of the analysed social group, and explanatory for detecting and describing the aspects and interrelationships that shape the study phenomenon. The focal group technique has been used.

### 2.2. Sample, Participants, and Context

The study population was the students’ parents of public secondary education high schools (SEHS) of Huelva, capital and province. The participants have been intentionally chosen based on the following selection and segmentation criteria:

- Having a child in any secondary education, higher secondary education or professional training course in any public centre of Huelva and its province.
- Socio-economic level of the family.
- Level of studies of the parents.
- Place of residence: rural area/urban area.
- Gender.

The participants’ recruitment was carried out through key informants linked to the educational and social fields. Potential participants were contacted by phone in advance to explain to them the purpose of the study and the work dynamics. Many denials of collaboration were produced from the extensive initial list of potential applicants, due to low availability. The socioeconomic level conditions health-related practices and beliefs. For this reason, different homogeneous groups were formed according to their social status [11], taking special care in creating heterogeneous groups considering the remaining inclusion criteria. Finally, the sample was made up of 42 people (Table 1). All participants who confirmed their attendance eventually attended, except for four of them. Prior to the start of each session, all informants confirmed their willingness to participate through informed written consent.

### 2.3. Data Collection

Five focus groups were created during a session lasting approximately 80 minutes. A piloted question guide was designed, after which appropriate adjustments and corrections were made (Table 2). The sessions were held in different spaces, adjusting to the comfort and accessibility of the participants. All groups were led by a moderator and an observer, both unrelated to the participants. All sessions were recorded in audio and two of them on video, with the permission of the informants. The transcript of the information was made after each session and was reviewed by two researchers, obtaining the saturation of the information after the fourth focus group. However, it was decided to schedule one more group in order to meet the established targeting criteria.

### 2.4. Data Analysis

The data analysis was carried out by three researchers, manually and with the support of the Atlas.ti programme, in several stages: 1. Repeated reading of literal transcriptions; 2. Setting up codes; 3. Building-up categories; 4. Comparing and discussing the results analysed by the researchers (triangulation) and agreeing on the final categorisation.

### 2.5. Ethical Commitments and Informed Consent

The research project in which this study is included was approved by the Bioethics Committee of the University of Huelva and has followed the principles of Law 14/2007 on Biomedical Research. The informed consent of all participants was obtained after receiving communication of the guarantee of their anonymity, and their free, voluntary and confidential participation.

## 3. Results

**Figure 1:**
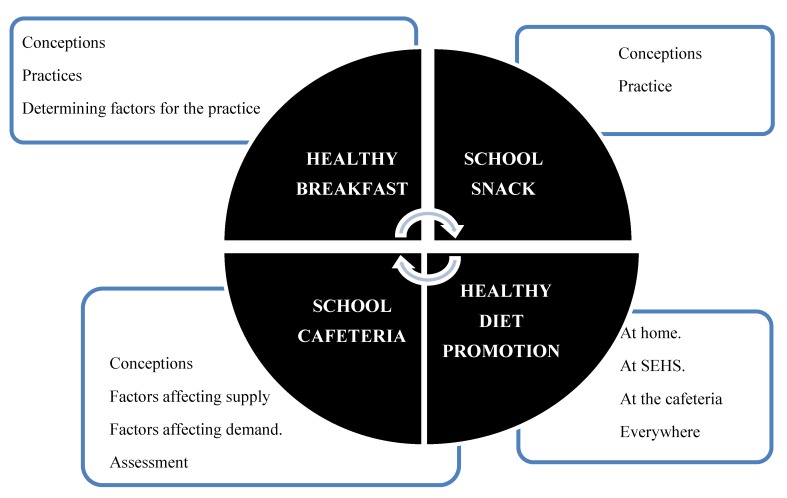
Study categories. Source: Self-prepared; 2017.

Four main categories were obtained from the triangulated analysis. Healthy Breakfast (HB), School Snack, School Cafeteria, and Measures for the Promotion of Healthy Eating Habits. Within them, subcategories were established, described in Figure 1. Evidence of the results is shown in Table 3, Table 4 and Table 5.

Regarding the Healthy Breakfast (HB) category (Table 3), informants had a clear and correct conception of it, considering as a full breakfast one that includes fruit, cereals and dairy products, and valuing breakfast as the most important meal of the day (B1–B4). The characteristic of being varied is as much or more appreciated than being complete (B5).

In the group which comprises participants with a lower socio-economic level, we do find confusion regarding the concept of a HB, indicating as complete types of breakfasts those where there was always a food group missing (B6–B7).

In the participants’ speeches, there emerges the realisation that the problem lies not in the misconception of a HB, but in its daily practice. (B8–B9). All family members made incomplete breakfasts, most often consuming a liquid dairy product (B15–B17). Participants also noted the variability in breakfast types that was practised within the family nucleus, finding a child who adopted a healthy model versus another one who ate an incomplete breakfast (B18–B20). The absence of breakfast seems to be motivated, above all, by the late awakening of schoolchildren, laziness, and lack of appetite in the mornings (B20–B22). Among the factors that determine this practice are the lack of time and comfort (B23–B27), since the realisation of a full breakfast requires more elaboration; lack of appetite in the mornings and temptations at home (B28–B30); informants highlighted the families’ lack of responsibility in creating good eating habits starting from childhood and promoting them in adolescence (B31–B34); the influence of the market (B35), where fast food advertising is quite powerful; and the pace of life of our society, which to a large extent they seem to blame on the great social pressure that is perceived.

The informants rated better habits at weekends, where they found it easier to carry out a HB, given the absence of haste, the presence of tranquility, or the possibility of sitting down for breakfast as a family (B10–B14).

As for the category of School Snack (Table 4), the participants’ conceptions and their practice are quite similar, being the star snack a sandwich (with omelette, mortadella, salami, pate...) and a drink, that is packaged juice or any dairy product (S1–S3).

During the talks, sadness was detected at the loss of healthy habits they had previously acquired in primary education, longing for the control of the centres (S5–S9). Participants attributed this loss of habit to the influence of peers in adolescence.

Regarding school cafeterias (Table 4), the informants expressed a dubious knowledge of their offer (C1). This was based on sandwiches, sweets, candy, and pizzas, and they coincided in identifying the omelette sandwich as the star product.

The influential factors regarding the cafeteria supply that emerged in the sessions were: the preferences and demand of adolescents; the influence of peripheral establishments, which they blamed for the consumption of fried products and sweets in schools (C2–C5); money management; the influence of peers and shame; time; and advertising in demand (C6–C13). Adolescents consider themselves and want to behave as adults, and parents give them greater confidence, offering them freedom to do their money management and to consume whatever they want during break.

The cafeteria rating was varied, depending on the participants. On the one hand, there were those who saw it as a service only for teachers (C14–C15). Others valued it better, for the comfort it entails for parents to alleviate morning preparation tasks (C16–C19). The greatest value to the cafeteria was given to those mothers belonging to the Association of Parents of Students (AMPA, for its acronym in Spanish), who take care of the school cafeteria, for the benefits of this activity.

In the category “Measures for the Promotion of Healthy Eating” (Table 5), implementation actions emerge at home: avoiding food temptations; improving lifestyle habits, such as dining a bit earlier and getting up earlier; getting children used to healthy eating; providing them with more fruits and healthy foods at breakfast; adults setting an example with healthy practices (S3–S7); not providing them with money, or providing them with the exact amount for the sandwich (S1–S2).

Among the cafeteria measures, they reflected on the teaching function this service has and, on the requirement, to act as a promoter of a healthy diet, with initiatives of placing fruit trays or giving away a piece of fruit when selling a sandwich (S16–S17). The “Fruit Day/Week” was an initiative created to promote the consumption of fruit, and offering other healthy products such as toasts, natural juices, daily homemade sweets, and the prohibition of selling unhealthy products, such as processed baked goods, packets of fried products, or candy (S18–S23). They stressed the need to return to the values instilled in primary education centres that regard eating habits during the school day, by establishing in the SEHS a snack system similar to that of primary education. They were convinced of the importance of the teachers’ and family’s involvement. An active approach towards promoting a healthy diet was not widespread among the teacher staff, nor was it among the families, who were not very involved. They called for the imposition of compliance and the prohibition of consuming candy, fried products, or processed baked goods at the centre (S9–S15).

There was great consensus to highlight the importance of education for the acquisition of healthy habits, and the responsibility and control on the part of the family (S24–S28).

## 4. Discussion

In order to cover the variability of the study unit, the development of focus groups with various types of families was considered. The diversity of the participants led to a higher participation in some groups, generating more information. Despite all this, the results were very similar in the different sessions.

Comparing our results with the existing literature has allowed us to find matches in aspects such as: the leading role of mothers in adolescent feeding [3], which is reflected in this study in the gender profile of the participants, given the difficulty of finding fathers who agreed to participate; the existence of knowledge, but its limited application in actual practice [27]; the absence of breakfast among adolescents [26], motivated by lack of hunger in the morning [23]; the detachment from the healthy eating model, a low intake of dairy and fruit, and a high intake of empty calorie-rich foods [4], especially in school snacks [8,10,11,19]; identification of support, family involvement, lack of healthy food at home and the establishment of standards as influential factors in the frequency of breakfast [22,24]; the highest prevalence of a HB during weekends [21]; and the relationship of the type of food intake during the school day, with the presence of establishments near the SEHS [8,33,34].

Most of the examined studies relate a higher educational, economic and social level to healthier eating and healthier breakfast [4,16,19,21,27], and not having breakfast or dinner as a family with an increased consumption of unhealthy food [13,14,15,26]. The importance and influence of the family in the quality of breakfast and food in general is closely related [12,14,22,27], coinciding with what was extracted from our analysis. The variability in the types of breakfast practiced within the family itself makes us reflect on the family educational models and the specific characteristics of the human being, that differentiate us, even at such early ages.

Families seem to challenge the services offered at school cafeterias. This fact, together with previous outcomes regarding their offer, which is categorised by low quality [6,8], makes us wonder whether this service is really desirable within the school context.

Another fact that deserves our reflection is the family’s ignorance of what the students consume during the school day, giving very poor value to food during this time slot.

## 5. Conclusions

Families are well aware of the composition of a HB, although it is not reflected in actual practice. Its absence is highlighted in many cases, as well as its variability in actual practice, according to family members. The star school snack was the sandwich, highlighting the families’ feeling of sadness, due to the loss of healthy habits acquired in primary education. There is a dubious knowledge of what is offered in the cafeteria and a diversity of opinions as to its true usefulness.

Regarding the difficulties that families encounter for their children to acquire healthy practices at breakfast and school snacks, the lack of time or comfort was mentioned, as well as the influence of their peers and the offer of the market.

As improvement proposals, the maintenance of healthy habits and the responsibility and control on the part of the family were encouraged. Additionally, there was a promotion of fruit and healthy products consumption from the part of the centre and its cafeteria, and proposals for various promotional activities, as well as the prohibition of consuming and selling processed baked goods, packets of fried products and candy in schools.

This study portrays the difficult reality families face in the acquisition and maintenance of healthy eating habits during the school day and allows us to identify future lines of action, so as to find alternative solutions to this reality. One example could be the control and responsibility of the main administrations, that is, estate, autonomic and educational administrations, to avoid selling products that are not recommended in a healthy diet at the school cafeterias, as well as the protection of advertisement campaigns aimed at children, to support the families by providing them with the necessary tools and resources, such as training in food choice and preparation abilities, parents’ control, etc.

In previous studies, no proposal has been made by families regarding the improvement of healthy eating habits among the youth during the school day. That is why we consider that this study provides something new, as it involves the direct opinion of families. The importance of the family in the acquisition and maintenance of healthy habits in general, and especially in their sons and daughters’ eating habits, justifies the need for interventions in this regard, promoting and encouraging the inclusion of healthy habits as real assets.

This study is part of a wider line of research in which eating habits were also assessed regarding Andalusian students. The results justify the need to assess the family context (presented in this article) and also the school context, so as to find solutions from a research perspective, as well as involving direct action and participation.

These actions may not only be aimed at students, or even families, but also administrations and the school context, and shall be taken as a joint commitment. An integrated model is sought, in order to imply the different sectors. Thus, our objective is to offer an inter-sector proposal to approach the issue from a global perspective that involves all the educational agents, by offering solutions from the different sectors, that is, families, educational, health, and social actors.

As for the limitations, a low level of participation on the part of parents was recorded in some groups. To avoid information bias, the groups were made up by choosing different family types.

## Figures and Tables

**Table 1:** Structure and Profile of the focal groups’ participants.

Group	Total	Gender	Number of Children in Secondary Education	Level of Studies	Socioeconomic Level	Type of Area of Residence
1	11	M (3) W (8)	1 (6) 2 (5)	Primary (3) Secondary (4) University (4)	Middle (7) Middle-high (4)	Urban (8) Rural (3)
2	6	W (6)	1(5) 3(1)	Primary (5) Secondary (1)	Middle-low (5) Low (1)	Rural (6)
3	9	M (1) W (8)	1(7) 2(2)	No studies (1) Primary (2) Secondary (3) University (3)	Low (1) Middle-low (5) Middle (1) Middle-high (2)	Rural (9)
4	9	W (9)	1(9)	No studies (5) Primary (3) Secondary (1)	Low (9)	Urban (9)
5	7	W (7)	1(4) 2(2) 3(1)	Secondary (4) University (3)	Middle-low (1) Middle (3) Middle-high (3)	Urban (5) Rural (2)

Source: Self-prepared; 2017/2018.

**Table 2:** Focal group questions script.

Question 1	What do you understand as a healthy breakfast?
Question 2	Which difficulties do you encounter for your children to acquire healthy habits during breakfast before leaving for the educational centre?
Question 3	What do your children often bring for breakfast at school? Do you regard it as healthy?
Question 4	What is offered at the school cafeteria your children attend? Do you consider the offer appropriate? Why?
Question 5	When encouraging your children to acquire healthy eating habits and, therefore, to choose a healthy snack at the school cafeteria, what limitations do you encounter?
Question 6	Which aspects could be improved at the school cafeteria? Which initiatives could be promoted?
Question 7	Do you consider the use of a school cafeteria necessary? Why is it useful for you?
Question 8	How can families contribute to improve healthy eating habits during the school day?

Source: Self-prepared; 2017.

**Table 3:** “Healthy Breakfast” study category.

Conceptions	B1 “There always must be natural fruit juice, some dairy products…. Cereal or toast, be it with margarine or olive oil…” (G1) B2 “The same for me, a healthy breakfast for me would include some bread, toast, some milk and some fruit” (G5) B3 “I think a piece of fruit, a glass of milk and some bread, whole-grain biscuits or something like that” (G2) B4 “Breakfast is very important because it can be considered the meal which will basically give the strength for the whole day due to the effort they make at school during the morning” (G1) B5 “For me, healthy is varied, that is, one day having toast with olive oil, garlic, ham, another day with tuna …” (G5) B6 “Cereal and milk” (G4) B7 “I believe it is fruit” (G4) B8 “For me, a Good breakfast differs a lot from what we really eat at home” (G1) B9 “What I do differs from what I believe” (G2) B10 “At weekends, at least in our home, they eat better” (G5) B11 “They are relaxed, we place breakfast in front of them and they seem to enjoy it more because they are even willing to have breakfast” (G5) B12 “In my case, during weekdays, I am always in a rush, but at weekends, Saturday and Sunday, I make breakfast for them and we all three sit together to have it” (G1) B13 “My case is that I watch Americans in films and they are all sitting together before leaving for school. We don’t do this, not at all, I think none of us do it…” (G2) B14 “Americans do it because my cousins act like this, when they are there, they tell me: Aunt, the cousins eat like in films” (G2)
Practice	B15 “I try to give them juice, dairies, and then anything I have at hand, something quick…” (G1) B16 “Chocolate powder, that’s it…” (G1) B17 “A glass of milk, chocolate powder and milk, but not fruit” (G3) B18 “I have two cases, my daughter, who has her chocolate powder and that’s enough, and José Manuel, who has his chocolate powder, toast, and before leaving for school, he takes half a glass of orange juice” (G3) B19 “I’ve got two, and each of them have breakfast differently. One has a mug of chocolate powder with soaked whole-grain biscuits, like porridge, and the other one has chocolate powder with a sweet, any processed baked sweet” (G2) B20 “I’ve got two, one who takes everything perfectly, the orange juice and a bowl with cereal, milk with cereal, and the other one, who never has breakfast, she has never had it” (G2) B21 “My son, nothing, he leaves for high school on an empty stomach” (G3) B22 “No, my son neither” (G4)
Determining factors	B23 “It is much faster to take any processed baked good, open it and they eat it… there are super easy foods to eat…” (G1) B24 “It is easier to have a croissant or *Bollicao* (processed baked bun) than preparing a piece of toast or a juice” (G2) B25 “Hurry, maybe the problem is just we are always in a hurry…” (G5) B26 “Time” (G1) B27 “I also think it is laziness” (G2) B28 “Mine cannot eat anything just woken up” (G4) B29 “Also temptations…” (G1) B30 “Well. before we did not have the sort of junk food we have today at home” (G2) B31“But that’s because we have created the wrong habit. I am fully aware of this, of having worse and worse habits. When I was a child, my parents didn’t give me such junk food our kids eat today, and I am the one to blame for this” (G2) B32 “I think it’s habit, because I’m the type of person who needs to have breakfast when I wake up, I can’t help but doing it, but my husband, for instance, and my daughter, can be on an empty stomach until noon, when they have breakfast. It depends on the person and on getting used to bad habits” (G2) B33 “It’s a matter of habit” (G2) B34 “It’s all about our habits. Many things that prevent you from waking up earlier because we haven’t had enough rest” (G1) B35 “Society is very important; burgers, pizzas, advertising doesn’t sell healthy products, only the worst which on top, are yummy” (G2)

Source: Self-prepared; 2017–2018.

**Table 4:** Study categories: Schools snack and cafeteria.

School Snack	
Conceptions	Same as Practice
Practice	S1 “Mine a sandwich, a sandwich and a juice” (G3) S2 “My children, sandwich and juice” (G3) S3 “My son takes money and buys a sandwich, always a sandwich, nothing else, and sometimes biscuits or muffins” (G5) S4 “I give him a packet of biscuits or something like that” (G4) S5 “In primary school, I used to give him more fruit to take to school… an Actimel” (G1) S6 “My son has also changed since he is in… he doesn’t want any fruit either…” (G1) S7 “A yoghurt to school? Horrible!” (G1) S8 “This differs a lot from Primary School. All this thing about toasts... They just want to go out to the playground” (G3) S9 “Mine used to eat fruit at school, but he doesn’t anymore” (G4)
**School Cafeteria**	
Conceptions	C1 “We somehow know from what our children tell us…” (G1)
Supply factors	C2 “It is demanded… If nobody demanded it… if children want an omelette sandwich or a packet of chips… well…” (G3) C3 “If we took all sweets away, first, the establishment opposite the school will sell them. It would be ideal not to allow taking any processed baked goods from outside, but this is done, so we can’t help but offering them” (G2) C4 “Mine buys something near the high school. I am not sure of what it is, a sandwich or a sweet, I think…” (G4) C5 “What happens is that, maybe not in the cafeteria, but in the establishment outside the high school, where children can halt, they sell them” (G1)
Demand factors	C6 “When children bring money to school, they buy whatever they want, and their parents may not even know about it” (G2) C7 “Well, as they are already attending high school, they believe themselves older” (G4) C8 “Now, in high school, “how shaming, an apple or a banana!” (G5) C9 “There is also the influence of their peers, their classmates…” (G5) C10 “They are not responsible for their own health, not at all” (G5) C11 “It’s just they don’t have the necessary time for breakfast, they don’t even have a full half an hour to say, well, I would have a hot chocolate drink with a piece of toast, they don’t have time, I’m sure” (G5) C12 “Yes, in a half-an-hour break it’s not possible” (G3) C13 “Society is very important; burgers, pizzas, advertising doesn’t sell healthy products, only the worst which on top, it’s yummy” (G2)
Assessment	C14 “It is for teachers, because they can have a coffee, but for children… I’m not sure about the school cafeteria being useful…” (G4) C15 “They must have a cafeteria for teachers to have a coffee, and a piece of toast, of course” (G5) C16 “Well, to have a quick fix, it’s ok” (G4) C17 “Well, for… I don’t know, if you forget to bring something one day, a bottle of water…” (G5) C18 “If a mother was not able, on a specific day, or couldn’t prepare breakfast for the child, this is an option to have something in the middle of the morning…” (G3) C19 “It’s true that the school cafeteria is useful, because you don’t have to prepare anything… they can buy a sandwich and a juice there, to have a sandwich…” (G1)

Source: Self-prepared; 2017–2018.

**Table 5:** Study categories: Measures towards promoting healthy diet.

At home	S1 “Sure, the solution would be not giving them the euro for breakfast but prepare the sandwich at home and put it into their schoolbags...” (G5) S2 “Well, me, either not giving them the money, or giving them just the exact amount of money for the sandwich, because if the sandwich is one euro, it’s just one, not one and 50 cents…” (G5) S3 “That they would eat more fruits…” (G4) S4 “If we did the same, that is, setting up an example” (G3) S5 “I believe that for children to eat fruit, parents need to do the same, and wake up a bit earlier to be able to have a relaxing breakfast” (G3) S6 “We have to set up an example, but I’m the type of person who cannot have breakfast when I just woke up” (G2) S7 “We have to get up a bit earlier, that is, you sacrifice and get up earlier and go to bed earlier” (G1)
At the centre	S8 “The PE teacher has a very Good technique for children to eat more fruit: if they have PE two days a week, and they take a piece of fruit, she gives them an extra point” (G1) S9 “High school teachers don’t help…” (G1) S10 “There are some that agree… but there are others who don’t want to and say they don’t have time…” (G2) S11 “Teachers could have any type of initiative, say, for example, one day a week, together with the cafeteria, promoting seasonal fruit such as strawberry, or Orange when the time comes, but always starting with support from school” (G3) S12 “To plan a weekly schedule with a sandwich on Mondays, fruit on Tuesdays, Wednesdays… As in Primary Education” (G3) S13 “To have a vending machine which sold fruit” (G4) S14 “At the bar… at least a tray with bananas… and apples, maybe” (G1) S15 “Look, for example, with the sandwich, you get a piece of fruit.” (G2) S16 “If the cafeteria is inside the school premises for other things, it should promote healthy diet as well” (G1) S17 “The cafeteria should be used to provide with training (how to make an omelette, healthy sandwiches…)” (G1)
At the cafeteria	S18 “Some sweets but homemade? The good old homemade sponge cake…” (G5) S19 “Just as they make omelettes, they could also make homemade sponge cakes, muffins…” (G5) S20 “They could offer fruit, toast with ham…” (G3) S21 “Having the “Week of the Fruit” every two months… something a bit healthier” (G3) S22 “Not selling candy. I understand there are all sorts of tastes, but there are healthy things that…” (G3) S23 “Maybe placing a table after the sandwich…there may be children who feel like having a piece of fruit or who haven’t had breakfast or don’t want a sandwich…placing a table with fruits and things like that…” (G2)
In general	S24 “I believe that what we create as habits for our children is what they are going to do later in their lives…” (G2) S25 “I believe education at home is the most important thing” (G5) S26 “As a mother, I think we always should have healthy things at home and that our children see us eating them and feel it’s what they should also do” (G1) S27 “I don’t even consider my daughter going to the school cafeteria, for anything at all” (G1) S28 “Diet responsibility and education are parents’ issues, so I believe that the key point is education, of course, about habits and control” (G1)

Source: Self-prepared; 2017–2018.
